# Supplementary material for: Highly Pathogenic Avian Influenza Viruses (HPAIV) Associated with Major Southern Elephant Seal Decline at South Georgia
Source: Commun Biol. 2025 Nov 13;8:1493. doi: 10.1038/s42003-025-09014-7 (PMC12615699; doi:10.1038/s42003-025-09014-7)
Supplement: Supplementary file 2 — Reporting summary [file 42003_2025_9014_MOESM2_ESM.pdf]

nature portfolio

Corresponding author(s): Dr Connor Bamford
Last updated by author(s): Sep 10, 2025

Reporting Summary

Nature Portfolio wishes to improve the reproducibility of the work that we publish. This form provides structure for consistency and transparency in reporting. For further information on Nature Portfolio policies, see our Editorial Policies and the Editorial Policy Checklist.

Statistics

For all statistical analyses, confirm that the following items are present in the figure legend, table legend, main text, or Methods section.

n/a	Confirmed	
☐	☒	The exact sample size (*n*) for each experimental group/condition, given as a discrete number and unit of measurement
☐	☒	A statement on whether measurements were taken from distinct samples or whether the same sample was measured repeatedly
☒	☐	The statistical test(s) used AND whether they are one- or two-sided<br>*Only common tests should be described solely by name; describe more complex techniques in the Methods section.*
☒	☐	A description of all covariates tested
☒	☐	A description of any assumptions or corrections, such as tests of normality and adjustment for multiple comparisons
☐	☒	A full description of the statistical parameters including central tendency (e.g. means) or other basic estimates (e.g. regression coefficient) AND variation (e.g. standard deviation) or associated estimates of uncertainty (e.g. confidence intervals)
☒	☐	For null hypothesis testing, the test statistic (e.g. *F*, *t*, *r*) with confidence intervals, effect sizes, degrees of freedom and *P* value noted<br>*Give P values as exact values whenever suitable.*
☒	☐	For Bayesian analysis, information on the choice of priors and Markov chain Monte Carlo settings
☒	☐	For hierarchical and complex designs, identification of the appropriate level for tests and full reporting of outcomes
☒	☐	Estimates of effect sizes (e.g. Cohen's *d*, Pearson's *r*), indicating how they were calculated

Our web collection on statistics for biologists contains articles on many of the points above.

Software and code

Policy information about availability of computer code

Data collection	eMotion v3.23
Data analysis	QGIS v3.22.16; Pix4D v4.9.0

For manuscripts utilizing custom algorithms or software that are central to the research but not yet described in published literature, software must be made available to editors and reviewers. We strongly encourage code deposition in a community repository (e.g. GitHub). See the Nature Portfolio guidelines for submitting code & software for further information.

Data

Policy information about availability of data

All manuscripts must include a data availability statement. This statement should provide the following information, where applicable:

- Accession codes, unique identifiers, or web links for publicly available datasets
- A description of any restrictions on data availability
- For clinical datasets or third party data, please ensure that the statement adheres to our policy

All UAV survey data are available on request from the Polar Data Centre or from the following DOIs: Data from the 2022/23 season for DPLUS109 are available for St Andrews Bay at https://doi.org/10.5285/f79b6577-d4cd-4eb2-9fec-b71e4e1d2389 & https://doi.org/10.5285/8189ed89-f36d-43c5-ae33-7e1c9ba0564d; for Hound Bay at https://doi.org/10.5285/eb210711-85e1-4671-97a8-8c2791979a19; and Gold Harbour at https://doi.org/10.5285/00cf7916-5c3f-42a2-8b98-38fa99b5aeb8.

Data for the 2024/25 season are available for St Andrews Bay at https://doi.org/10.5285/e238f84e-63b2-4019-8308-7398a8ea204f; for Hound Bay at https://doi.org/10.5285/85de16ff-a3b2-42b4-a898-aea94ee47b83; and for Gold Harbour at https://doi.org/10.5285/47d0718d-7146-44d3-965c-60e62a48b8cc.

Research involving human participants, their data, or biological material

Policy information about studies with human participants or human data. See also policy information about sex, gender (identity/presentation), and sexual orientation and race, ethnicity and racism.

Reporting on sex and gender	n/a
Reporting on race, ethnicity, or other socially relevant groupings	n/a
Population characteristics	n/a
Recruitment	n/a
Ethics oversight	BAS Animal Ethics review AWREB 1071 and 1109

Note that full information on the approval of the study protocol must also be provided in the manuscript.

Field-specific reporting

Please select the one below that is the best fit for your research. If you are not sure, read the appropriate sections before making your selection.

☐ Life sciences ☐ Behavioural & social sciences ☒ Ecological, evolutionary & environmental sciences

For a reference copy of the document with all sections, see nature.com/documents/nr-reporting-summary-flat.pdf

Ecological, evolutionary & environmental sciences study design

All studies must disclose on these points even when the disclosure is negative.

Study description	This work presents the first quantitative evidence of the impact of high pathogenicity avian influenza virus (HPAIV) on a key marine mammal species in the sub-Antarctic at their largest population hub, South Georgia. In 2024, we recorded a dramatic 46.6% (SD = 13.5%) decline in counts of breeding females at South Georgia's three largest southern elephant seal colonies following the arrival of HPAIV in 2023. Extrapolating from historical census data, this equates to 62,000 missing females, with likely long-term implications for population viability and ecosystem stability.
Research sample	Southern elephant seal (SES, Mirounga leonina) breeding colonies at Hound Bay, St Andrews Bay and Gold Harbour, South Georgia.
Sampling strategy	UAV aerial imagery collected over the three largest SES breeding beaches on South Georgia. These beaches represent ~ 15% of the island's population and were deemed to be representative of the islands population at the last census (Boyd et al. 1996).
Data collection	UAV aerial imagery were collected during the peak of breeding in 2022 and 2024, straddling the arrival of HPAI on the island in 2023. Manual counts of female seals visible in these images are then made by an experienced observer and reviewed by another in QGIS. Counts of female seals present during peak breeding times are indicative of overall population health, and are a long-established metric for measuring populations of this species (see references within).
Timing and spatial scale	2022: 17th October till 10th November. 2024: 17th October till 10th November. Comparisons between the counts made in 2022 and 2024 are made in the paper, and represent field effort in the year before and year after the arrival of HPAI on South Georgia.
Data exclusions	No data removal
Reproducibility	All raw imagery are available open access via the DOIs listed in the paper. Data from 2022 are under embargo until December 2025 and data from 2024 are under embargo until June 2026. Interested parties may contact the PDC or lead authors ahead of these dates to access data on a case-by-case basis. Counts of female seals were made by experienced field biologists familiar with the study species, who differentiated sex based on the extreme sexual dimorphism exhibited by this species. All attempts to process these data were successful. No lab-based or statistical experimentation was carried out or failed, respectively.
Randomization	Randomisation is not applicable to this study as the data consists of absolute counts made by an experienced observer (reviewed by another) of the total number of female seals present on comparable days two-years apart, straddling the emergence of HPAI on the island. Percentage change were then observed and reported.
Blinding	This is not applicable as there is not test condition/grouping for this work.

Did the study involve field work? ☒ Yes ☐ No

Field work, collection and transport

Field conditions	UAV flights during fair weather conditions (<10m/s windspeed, no rainfall) to collect overhead aerial imagery)
Location	Hound Bay (-54.37S -36.21W); St Andrews Bay (-55.44S -36.18W), and Gold Harbour (-54.63S -35.94W), South Georgia.
Access & import/export	Access to Hound Bay and St Andrews Bay was on foot following a small boats drop off from King Edward Point Research Station, and access to Gold Harbour was facilitated by the National Geographic Explorer. All work was conducted under Government of South Georgia and the South Sandwich Islands (GSGSSI) Regulated Activity Permit Numbers 2022/021 and 2024/028, and ASSI permits P/314 and P/444 & 445 for the 2022 and 2024 season, respectively. Work was approved by BAS' Animal ethics board permits 1071 and 1109 for 2022 and 2024, respectively.
Disturbance	All flights were conducted from a 'stand-off' distance, well removed from the breeding colonies. the UAV operated at ~90m as to avoid disturbance. Take-off and landing locations were selected as to be awa from any animals in line with BAS' Wildlife Interaction Handbook and GSGSSI RAP permits.

Reporting for specific materials, systems and methods

We require information from authors about some types of materials, experimental systems and methods used in many studies. Here, indicate whether each material, system or method listed is relevant to your study. If you are not sure if a list item applies to your research, read the appropriate section before selecting a response.

Materials & experimental systems

n/a	Involved in the study
☒	☐ Antibodies
☒	☐ Eukaryotic cell lines
☒	☐ Palaeontology and archaeology
☐	☒ Animals and other organisms
☒	☐ Clinical data
☒	☐ Dual use research of concern
☒	☐ Plants

Methods

n/a	Involved in the study
☒	☐ ChIP-seq
☒	☐ Flow cytometry
☒	☐ MRI-based neuroimaging

Animals and other research organisms

Policy information about studies involving animals; ARRIVE guidelines recommended for reporting animal research, and Sex and Gender in Research

Laboratory animals	This study did not involve lab animals.
Wild animals	This study involved no handling of wild animals. Overflight of southern elephant seal (Mirounga leonina) breeding colonies at the peak of breeding ~25th October in 2022 and 2024 to collect aerial imagery. These flights overflew all age demographics and also likely overflew the occasional Antarctic fur seal (Arctocephalus gazella) and portions of king penguin (Aptenodytes patagonicus) colonies.
Reporting on sex	Results presented here pertain to only counts of female elephant seals. Counts of female elephant seals are a well-established metric for monitoring populations of this species and are consistent with all comparisons made in the paper. Male elephant seals are not a limiting factor on elephant seal populations. Sex was determined by experienced observers reviewing the UAV imagery, aided by the extreme sexual dimorphism exhibited in this species, whereby males are 5-6 times larger than females by weight.
Field-collected samples	The study did not involve physical samples collected in the field. Aerial imagery were backup onto multiple hard drives and carried out of the field by the team.
Ethics oversight	Animal ethical approval was reviewed and granted by BAS' Animal Ethics board for both fieldwork seasons (AWERB Permits 1071 and 1109).

Note that full information on the approval of the study protocol must also be provided in the manuscript.

Plants

Seed stocks	n/a
Novel plant genotypes	n/a
Authentication	n/a
